# Fabrication of monodisperse magnetic nanorods for improving hyperthermia efficacy

**DOI:** 10.1186/s12951-021-00794-8

**Published:** 2021-03-01

**Authors:** Shan Zhao, Nanjing Hao, John X. J. Zhang, P. Jack Hoopes, Fridon Shubitidze, Zi Chen

**Affiliations:** 1 Thayer School of Engineering, Dartmouth College, 14 Engineering Drive, Hanover, 03755 NH USA; 2 Geisel School of Medicine, Dartmouth College, 1 Rope Ferry Road, Hanover, 03755 NH USA; 3 Division of Thoracic Surgery, Brigham and Women’s Hospital, Harvard Medical School, Boston, 02115 MA USA

## Abstract

**Background:**

Hyperthermia is one of the promising cancer treatment strategies enabled by local heating with the use of tumor-targeting magnetic nanoparticles (MNP) under a non-invasive magnetic field. However, one of the remaining challenges is how to achieve therapeutic levels of heat (without causing damages to regular tissues) in tumors that cannot be effectively treated with anti-tumor drug delivery.

**Results:**

In this work, we report a facile method to fabricate magnetic nanorods for hyperthermia by one-step wet chemistry synthesis using 3-Aminopropyltrimethoxysilane (APTMS) as the shape-controlling agent and ferric and ferrous ions as precursors. By adjusting the concentration of APTMS, hydrothermal reaction time, ratios of ferric to ferrous ions, magnetic nanorods with aspect ratios ranging from 4.4 to 7.6 have been produced. At the clinically recommended field strength of 300 Oe (or less) and the frequency of 184 kHz, the specific absorption rate (SAR) of these nanorods is approximately 50 % higher than that of commercial Bionized NanoFerrite particles.

**Conclusions:**

This increase in SAR, especially at low field strengths, is crucial for treating deep tumors, such as pancreatic and rectal cancers, by avoiding the generation of harmful eddy current heating in normal tissues.
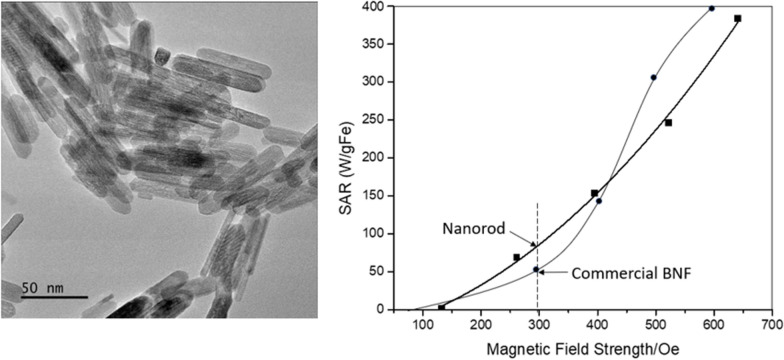

## **Background**

Hyperthermia is one of the conventional methods in cancer therapeutics, whose efficacy, however, is limited by the relatively low heating efficiency. Hyperthermia can be induced in tumor tissue by embedding magnetic nanoparticles and activating them with an alternating magnetic field and cell death can occur when a sufficient cumulative effective heat dose is reached [1–4]. The treatment can be targeted to the tumor area by accurate placement of the nanoparticles and targeting the activating magnetic field [5, 6]. Compared with chemotherapy and radiotherapy, targeted hyperthermia therapy has the potential to have fewer systemic side effects and less damage to the surrounding normal tissue [7–11]. In the past, a limitation for magnetic hyperthermia is the difficulty of generating sufficient heat in the tumor area with a biologically safe AMF (alternating magnetic field). Even with direct injection into the tumor, it is difficult to deliver a sufficient thermal dose with reasonable injected MNP volumes [2, 7, 9]. In each environment, the heating achieved is determined by the amount of iron in the target area and the watts per gram (SAR/specific absorption rate) of heat created by that iron when it is exposed to an alternating magnetic field [12–14]. AMF field strengths are limited by safety concerns over eddy current heating and tissue uptake can limit iron concentrations in the tissue. Therefore, any increase in the SAR of the iron present in the target area would be advantageous. In order to achieve an efficient heat treatment with minimal normal tissue risk for cancer patients, it is imperative to develop new magnetic nanomaterials with high heating efficiency or SAR at the lowest particle dose [15–17]. Since, the SAR value strongly depends on the intrinsic properties including saturation magnetization (M_s_) and geometric anisotropy of the nanoparticles [18], one effective method to improve SAR is to increase the nanoparticle anisotropy and M_s_ by modifying their shape and/or size [15, 19].

Anisotropic one-dimensional (1D) nanostructured materials (nanotubes, nanorods and nanowires) are favorable due to the restricted carrier motion in two directions, high surface to volume ratio, and a proven increase of selective attachment to specific organs [20]. 1D iron oxide materials, such as nanorods, nanotubes, nanowires and nanobelts, are found to have potential applications in medical, electrical, magnetic and optical nanodevices [21]. The most common iron oxide crystallites include hematite (α-Fe_2_O_3_), maghemite (γ-Fe_2_O_3_), goethite (α-FeOOH), akageneite (β-FeOOH or Cl-FeOOH), and magnetite (Fe_3_O_4_).

Due to its unique superparamagnetic properties, magnetite (Fe_3_O_4_) offers excellent potentials in biomedicine. These potentials include magnetic separation, therapeutic drug delivery, radio frequency methods for tumor ablation via hyperthermia and contrast enhancement agents for magnetic resonance imaging [22]. Previous researchers [23–25] have confirmed the biocompatibility of magnetite, which makes it a very promising candidate for biomedical applications. A wide variety of magnetite nanorods have been developed: Vayssieres et al. [26] have developed an aqueous chemical growth of oriented three-dimensional crystalline nanorod arrays of Fe(III) oxides; Ramírez and co-workers [27] reported synthesis of a high magnetite fraction in superparamagnetic nanospheres by a three-step mini-emulsion polymerization; Ding et al. [28] prepared magnetite nanorods in the presence of PANI nanorods; Zhang et al. [29] reported new nanorods using reverse co-precipitation technique with external magnetic field. In the previous works, synthesis of spindle magnetic oxide needed multi-steps of processing. Although Wan et al. [21] and Chen et al. [30] reported two facile approaches to fabricate Fe_3_O_4_ nanorods with the length ranging from 200 to 2000 nm, they were non-uniformly dispersed. The agglomeration of these particles originates from the strong interaction among the particles, which will diminish their magnetic properties [28]. Si et al. [31] presented a solvothermal method to prepare single crystal water-phased Fe_3_O_4_ nanorods with a tunable aspect ratio (length from 58 to 250 nm, width from 8 to 64 nm). Das [32] designed a crystalline Fe_3_O_4_ nanorod with a tunable aspect ratio, which showed significantly enhanced SAR values compared to their spherical and cubic counterparts. They also found that increasing the aspect ratio of the nanorods from 6 to 11 could improve SAR by 1.5 times. Most recently, Salvador et al. designed superparamagnetic magnetite nanoparticles of the size between 5.4 and 7.2 nm using a W/O microemulsion system, which could be advantageous in effectively controlling the size, shape, and composition of the nanoparticles [33]. Konopacki et al. indicated that the rotating magnetic field can serve as a reinforcing factor on the heating distribution of nanoparticles (GO, Fe_3_O_4_) and hybrid material (GO/Fe_3_O_4_), and also showed the promise of using hybrid material as an effective hyperthermia agent [34].

As one of the iron oxide crystals, akaganeite has the less common bcc packing rather than the hcp packing in goethite and hematite [35]. The octahedra with face-shared double chains of (FeO_6_)^−6^ would propagate along fourfold symmetrical b-axis, which offers an open tunnel lattice structure to accommodate anionic species [36]. The tunnels turn parallel to the b-axis and are delineated by (110) plane. Unlike goethite that is stabilized by integrating with OH^−^, akaganeite is usually stabilized by F^−^ or Cl^−^ [35]. Crystallographically, akaganeite exhibits a hollandite-like structure with monoclinic symmetry [35], and it tends to grow into crystalline 1-D irregularly shaped, ellipsoidal nanostructure [37]. Therefore, akaganeite can serve as the intermediate phase for the nucleation of the oxide phase, thereby playing a role in defining the final 1-D nanostructure [38].

In this work, we developed a direct 3-aminopropyltrimethoxy-silane (APTMS)-assisted hydrothermal method to fabricate magnetic nanorods with intermediate aspect ratio and enhanced magnetic heating properties. The effect of the reaction conditions, including the quantities of APTMS, hydrothermal reaction time, ratios of Fe(II) to Fe(III) ions, on the morphology of magnetic nanorods and the hyperthermia response of nanostructures was identified. The probable nanorod growth mechanism from intermediate akaganeite phase and the heating mechanism were further discussed in this paper.

## Results

### Characterizations of magnetic nanorod

**Fig. 1:**
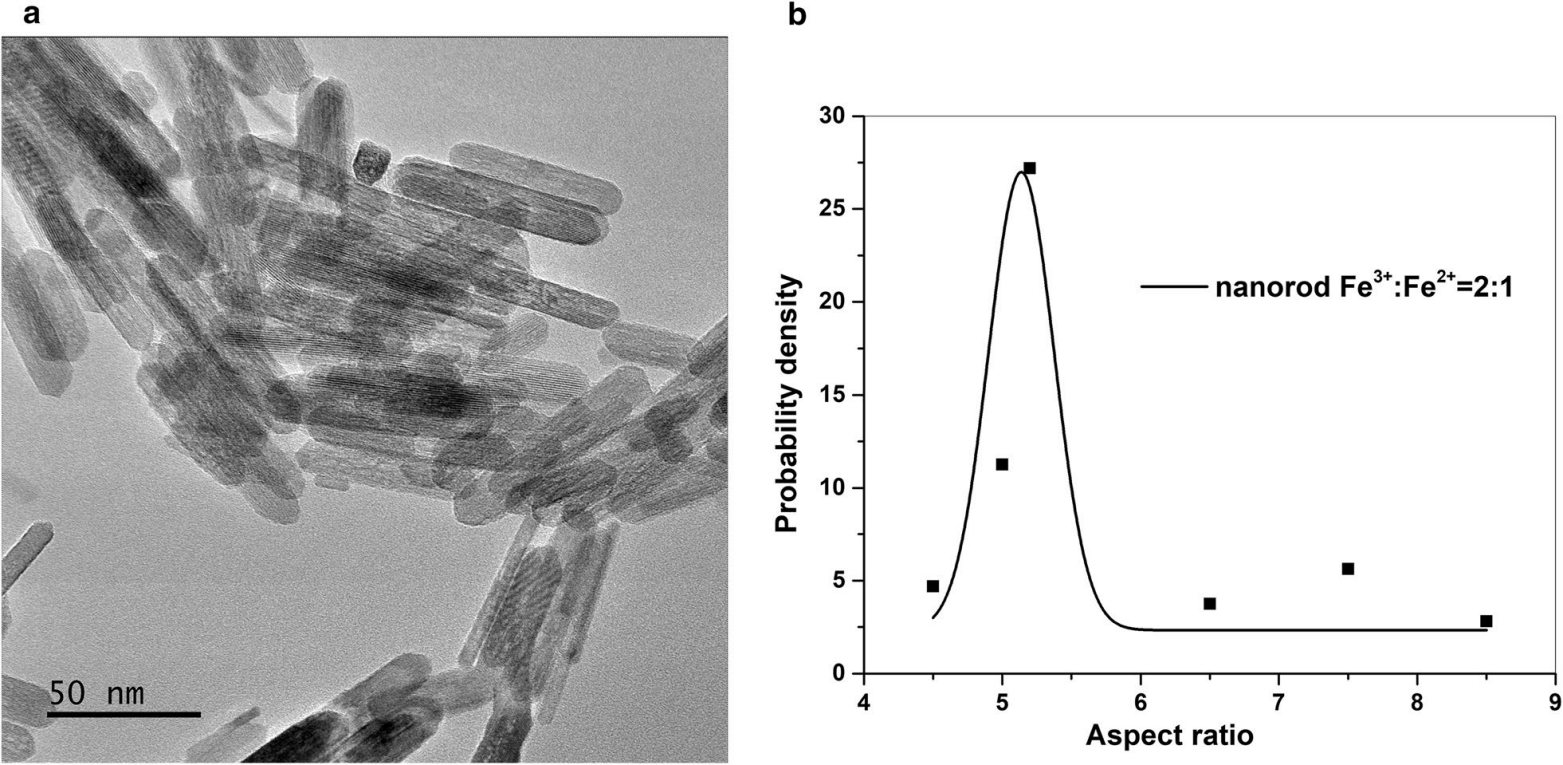
TEM image of magnetic nanorod with Fe^3+^/Fe^2+^=2:1 (hydrothermal for 3 h) and derived aspect ratio probability density (**b**)

Representative TEM micrograph of nanorods is presented in Fig. 1a. Using TEM imaging, we are able to show the structure and size distribution (range 50–70 nm in length and 8–10 nm in width) of the nanorods. The average aspect ratio is 5.75 for these nanorods (Fig. 1b).

**Fig. 2:**
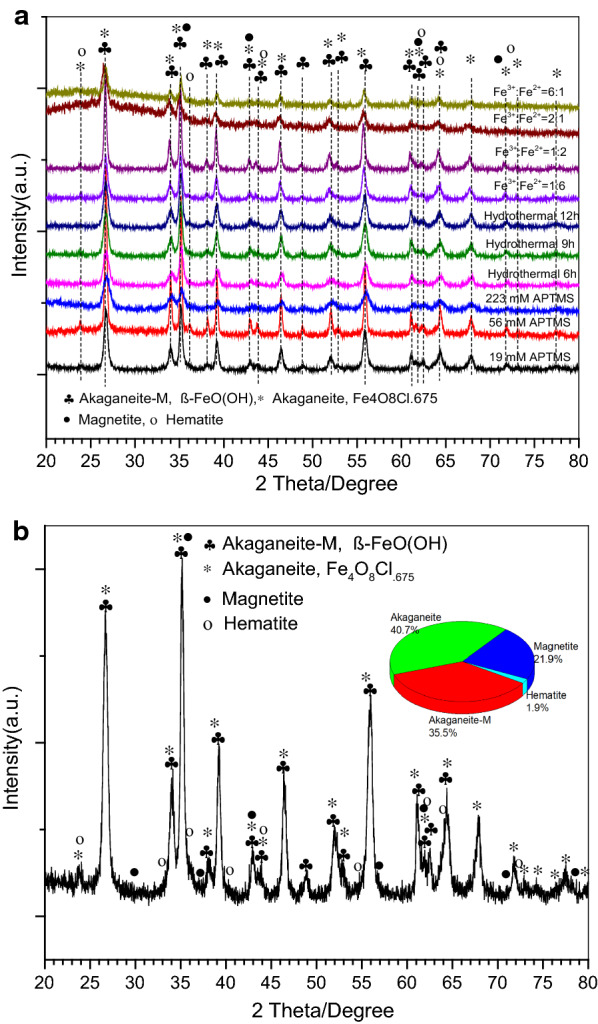
XRD patterns for all **a **and the representative nanorod sample made of Fe^3+^/Fe^2+^=2:1 during hydrothermal reaction for 9 h (**b**)

X-ray diffraction (XRD) is used to determine the purity and phase structure of nanoparticles. The XRD pattern (Fig. 2a) confirms the coexistence of the akageneite-M (space group: *tl4/m*), akageneite (*Ml2/m*), magnetite (*cFd-3m*) and hematite (*hR-3c*). The diffraction peaks indexed to the (110), (400), (440), (533), (444) planes imply the spinel cubic structure (space group: *Fd3m*), confirming the crystallization of the standard magnetite (JCPDS:19–0629) [39]. For the representative nanorod sample (Fig. 2b), the structural refinement indicates 21.9 wt% of magnetite and 1.9 % of hematite. The observation of hematite could also due to the drying process in air which transformed magnetite to hematite.

**Fig. 3:**
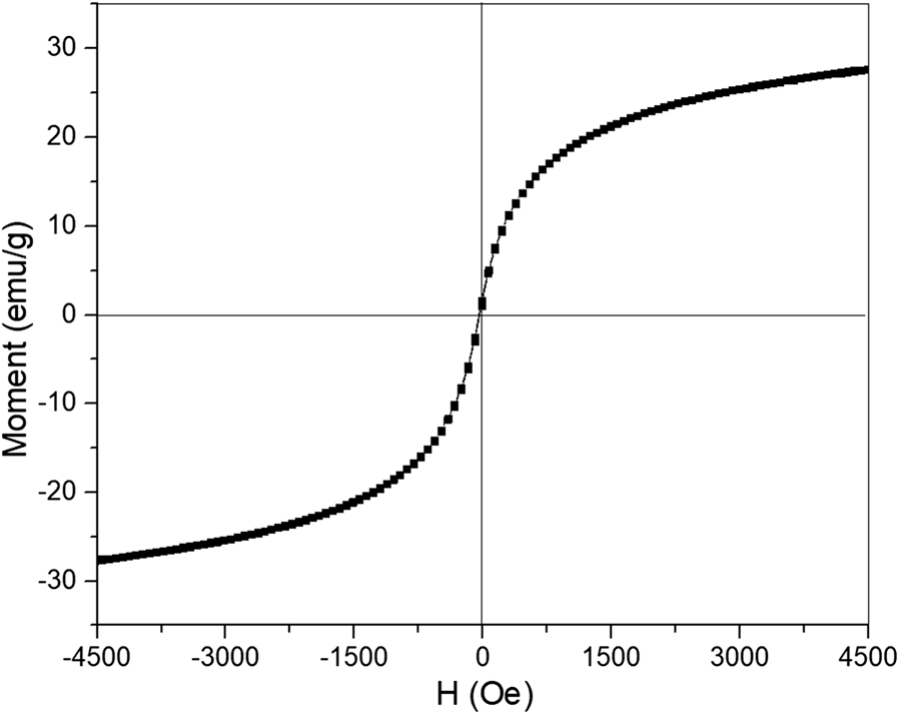
Magnetization versus magnetic field strength for magnetic nanorod with Fe^3+^/Fe^2+^=2:1 (hydrothermal for 9 h)

The magnetization curve of the representative nanorod sample shows superparamagnetic behavior with saturation magnetization of 28 emu/g (Fig. 3). The magnetization value of nanorod is much larger than the previous reported pure hematite alone [40, 41], which could due to the ferromagnetic nature of magnetite, of which the magnetization in pure form can reach up to 100 emu/g [42].

### Inductive heating properties of nanorod

**Fig. 4:**
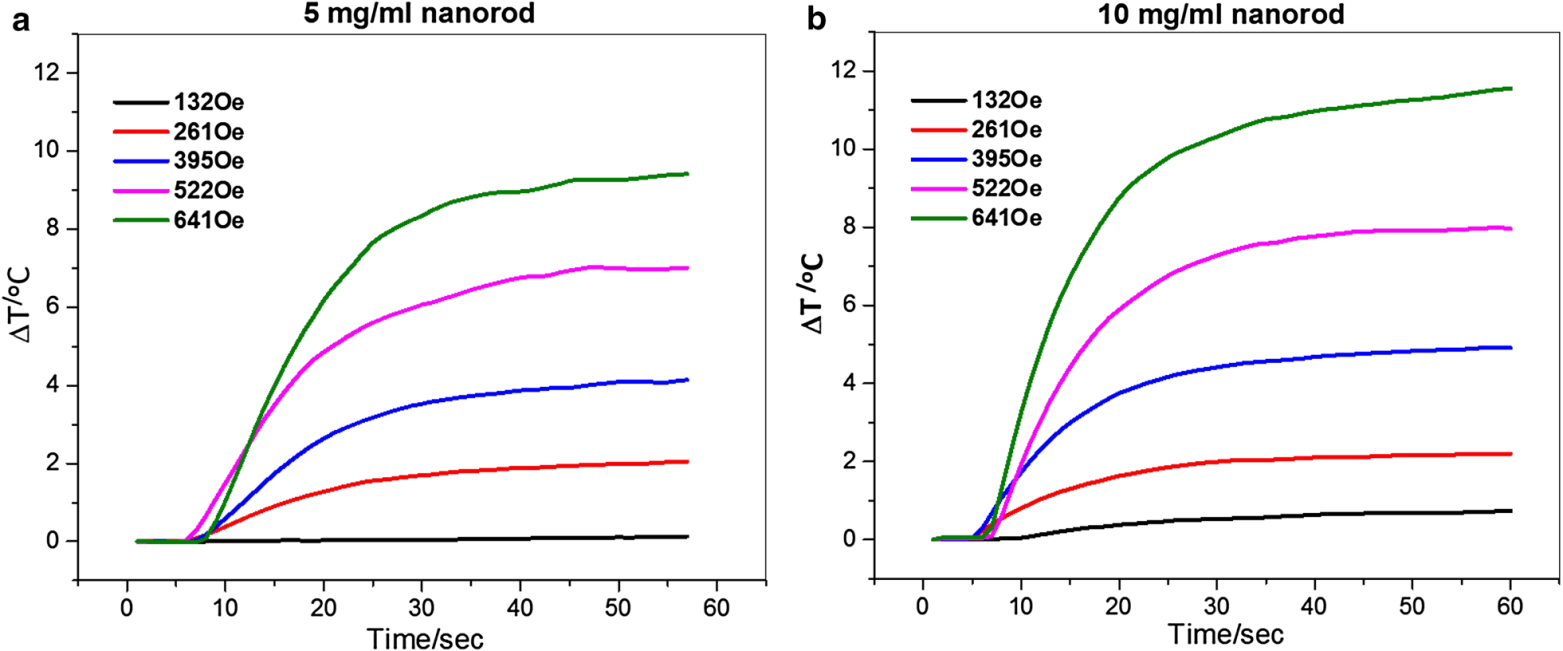
Temperature versus time for nanorod (with Fe^3+^/Fe^2+^=2:1, hydrothermal for 9 h) at different field strengths at a frequency of 184 kHz

We demonstrate in Fig. 4 that by changing the strength of the applied AMF field, and the nanorod concentrations, the final temperature reached can be controlled. At the tested AMF field strengths 261 Oe through 641 Oe, nanorods have been proven to produce more heat and with increasing the field strengths. In Fig. 4, we have plotted the heating curves of nanorods in water for two different concentrations, 5 and 10 mg/mL, respectively. The nanorod solution with a concentration of 10 mg/mL produced a temperature increase of 11.5 °C in 60 s at 641 Oe, while that with a concentration of 5 mg/mL produced a temperature increase of 9.5 °C over the same time period. Since no significant hysteresis was observed (Fig. 3), the field-driven viscous frictional loss could be a major source of the generated. At a low field strength of 132–385 Oe there is no obvious distinction in the heating efficiency between these two samples, whereas at a higher field strength of 522 or 641 Oe, the heating efficiency shows an observable variation depending on the iron concentration. This discrepancy implies that the loss of heat due to friction or resistance of viscosity (concentrations) in a low oscillating magnetic field is less prominent than that in a high field.

**Fig. 5:**
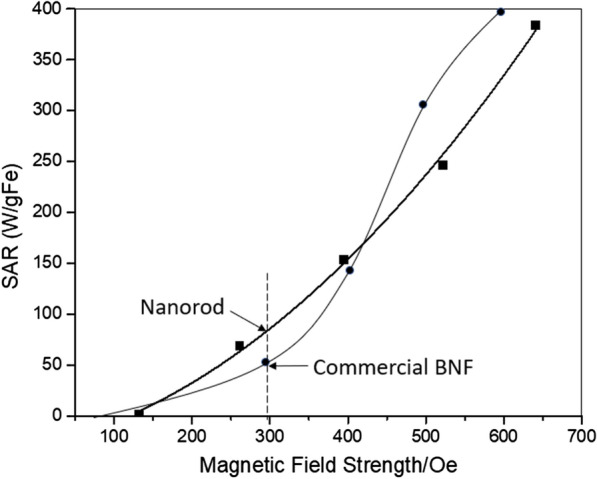
SAR for 5 mg/ml magnetic nanorod (with Fe^3+^/Fe^2+^=2:1, hydrothermal for 9 h) and commercial BNF particle at a frequency of 184 kHz. The SAR for BNF is given for comparison

 As shown in Fig. 5, under an AMF of 261, 395, 522 Oe at 184 kHz, nanorods of 5 mg/ml displayed the SAR (specific aspect ratio) values of 69, 154, 246 W/g respectively, which is approximately 50 % higher than that of commercial Bionized NanoFerrite (BNF) particles [43]. In this paper, we used the commercial Bionized NanoFerrite particles as a benchmark to our nanorod because we wanted to make sure that both group of those data are collected in the same instrument using the same setup, same frequencies and field strength. Since all SAR depends not only the material but also the testing parameters, such as frequencies and field strength, and the higher frequencies and field strength one uses, the higher SAR one can get for the same sample. Since the AMF-based hyperthermia (mNPH) system we used to test the SAR was custom built, the heating measurements were performed using a 14-turn, air core, copper solenoid coil (internal diameter 32 mm, length 12 cm), which was powered by a 10 kW TIG 10/300 generator (Hüttinger Elektronik GmbH, Freiburg, Germany) and cooled by running water kept at 20 ^o^C by a closed circuit chiller [43]. It is not easy to compare the magnetic properties between our nanorods and other magnetic nanomaterials since they were tested under different frequencies and field strength than ours. For example, most data of SAR shown in Table 1 from Hervault et al. [3] were tested at frequencies over 400 kHZ, while in our case we use 184 kHz, which is more clinically relevant; the only example listed in Table 1 [3] using 100 kHz and 377 Oe had an SAR of 29 W/g, which is far less than our value of 154 W/g at frequency of 184 kHz and field strength of 395 Oe. This increase in SAR especially in a low field strength (300 Oe or less, clinical recommended [44]) is crucial for treating deep tumors, such as pancreatic and rectal cancers. The AMF produced by a coil can penetrate inside tissue and activate heating of MNPs in tissues, meanwhile due to the AMF, the unwanted eddy currents in the normal tissues also occurs [45, 46]. Defined by Faraday’s law, the absorbed power density in tissue due to eddy currents is σ(πµ_0_*Hfr*)^2^ = σ*E*^2^, where *f* is the frequency, σ is the tissue conductivity, µ_0_ is the permeability of free space, *r* is the radial position within the solenoid in which the tissue exists, *E* is the electric field and *H* is the magnetic field [45]. Therefore, lowering field strengths could be an alternative to produce lower unwanted eddy current heating in surficial tissues.

### Influence of the APTMS concentrations

**Fig. 6:**
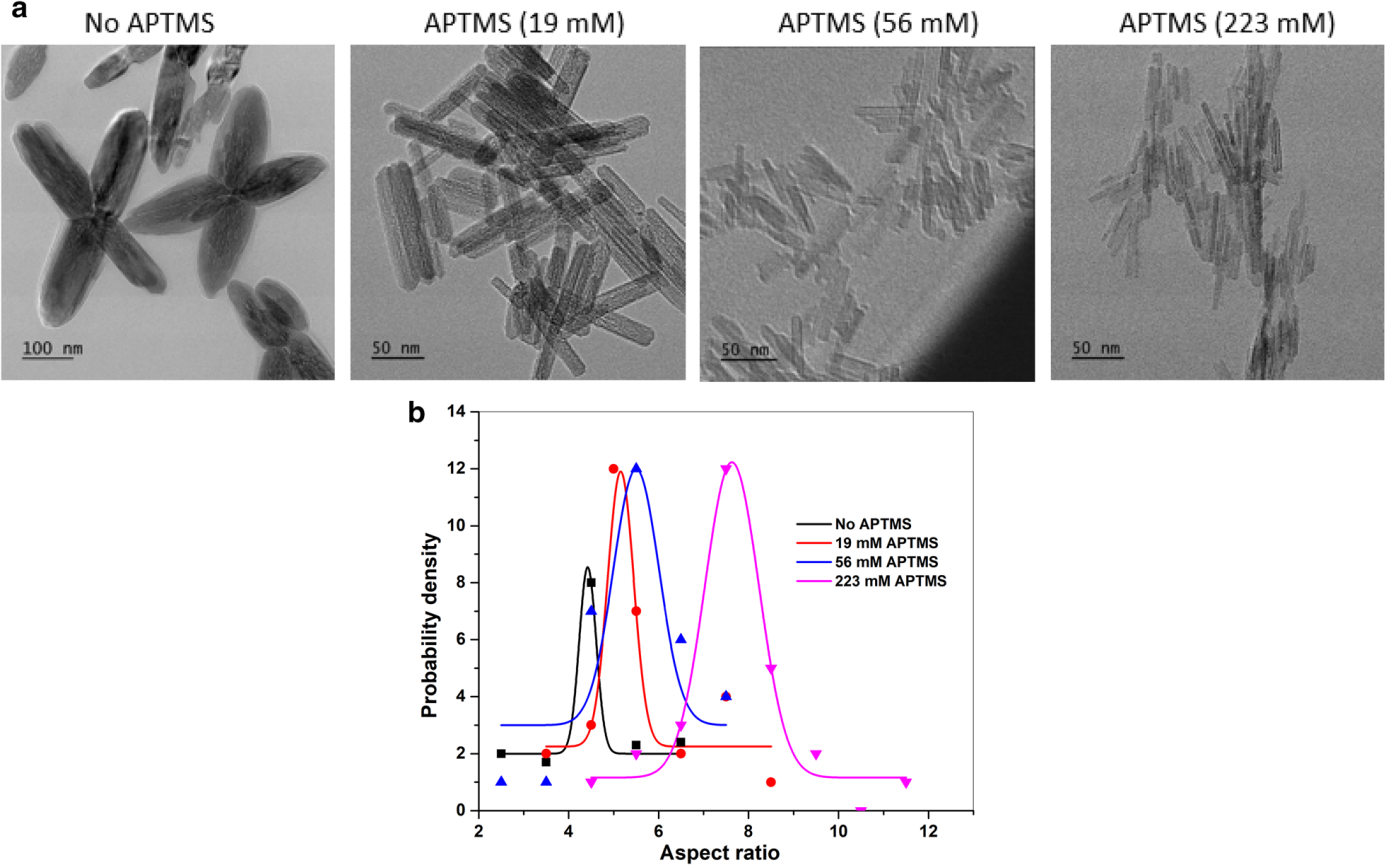
Representative TEM images **a** and aspect ratio probability densities **b** for nanorods with changing of APTMS from 0 to 1.2 g (Fe^3+^/Fe^2+^=2:1, hydrothermal for 12 h)

The TEM images in Fig. 6a demonstrate nanorods synthesized at different concentrations of APTMS, while keeping the ratio of Fe^3+^/Fe^2+^ (2:1) and hydrothermal time (12 h) constant. The grown nanorod crystals are seen to increase in aspect ratio with increasing concentrations of APTMS (Fig. 3), from 4.4 to 5.1 to 5.5 and 7.6, respectively. At low concentration of APTMS (0 mM ≤ [APTMS] ≤ 19 mM), an increased occurrence of crystallographic twinning of nanocrystals is seen as the four-pointed stars. The crystallographic twinning of nanocrystals grown from solutions could result from the (332) twin system with an angle of 62 ° [47]. The existence of small amount of Si (from APTMS) in β-FeOOH increases the twinning [47].

TEM images further confirmed the formation of nanorods with a narrow size distribution. The average dimension of the nanorods is 100 nm×20 nm, 65 nm×11 nm, and 80 nm×10 nm for different concentrations of APTMS (19, 56, 223 mM), respectively. Higher [APTMS] is shown to aid the more regular arrangement arrays of monodisperse nanorods along longitudinal directions. This may be linked to base nature of APTMS in water, which modulates the assembly pathway of the polymorphs nanorods in solution.

**Fig. 7:**
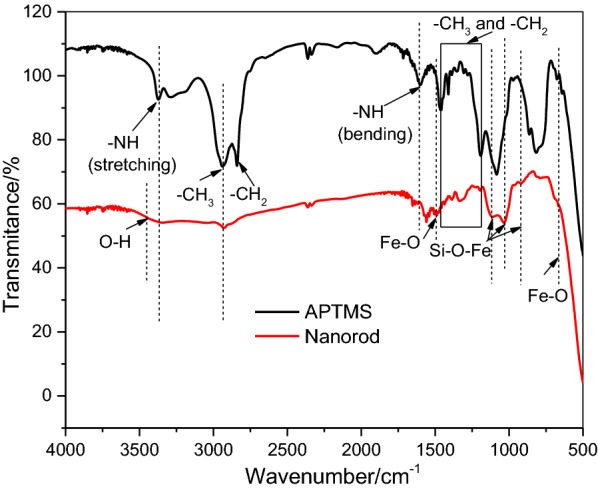
FTIR spectrum of nanorods

The IR transmittance spectrum provides surface chemistry of nanorod (Fig. 7). The bands at 3200–3550 cm^− 1^ due to the ɣ(O-H) vibration of H_2_O can be observed and this broadening is related to the presence of N-H stretching at 3378 cm^− 1^ [48]. The bands at 660 and 923, 1050–1250 cm^− 1^ are the characteristic Fe-O and Si-O-Fe vibrations [49, 50]. Additionally, the CH_3_ at 2939 cm^− 1^ and NH_2_ bending at 1599 cm^− 1^ substantiate the APTMS functionalization on iron oxide [48]. Several sharp peaks are observed in the range 1460 − 1113 cm^− 1^, due to the methylene (-CH_2_-) and methyl (-CH_3_) group bending and C-O stretching [51].

### Influence of the Fe^3+^/Fe^2+^ molar ratio

**Fig. 8:**
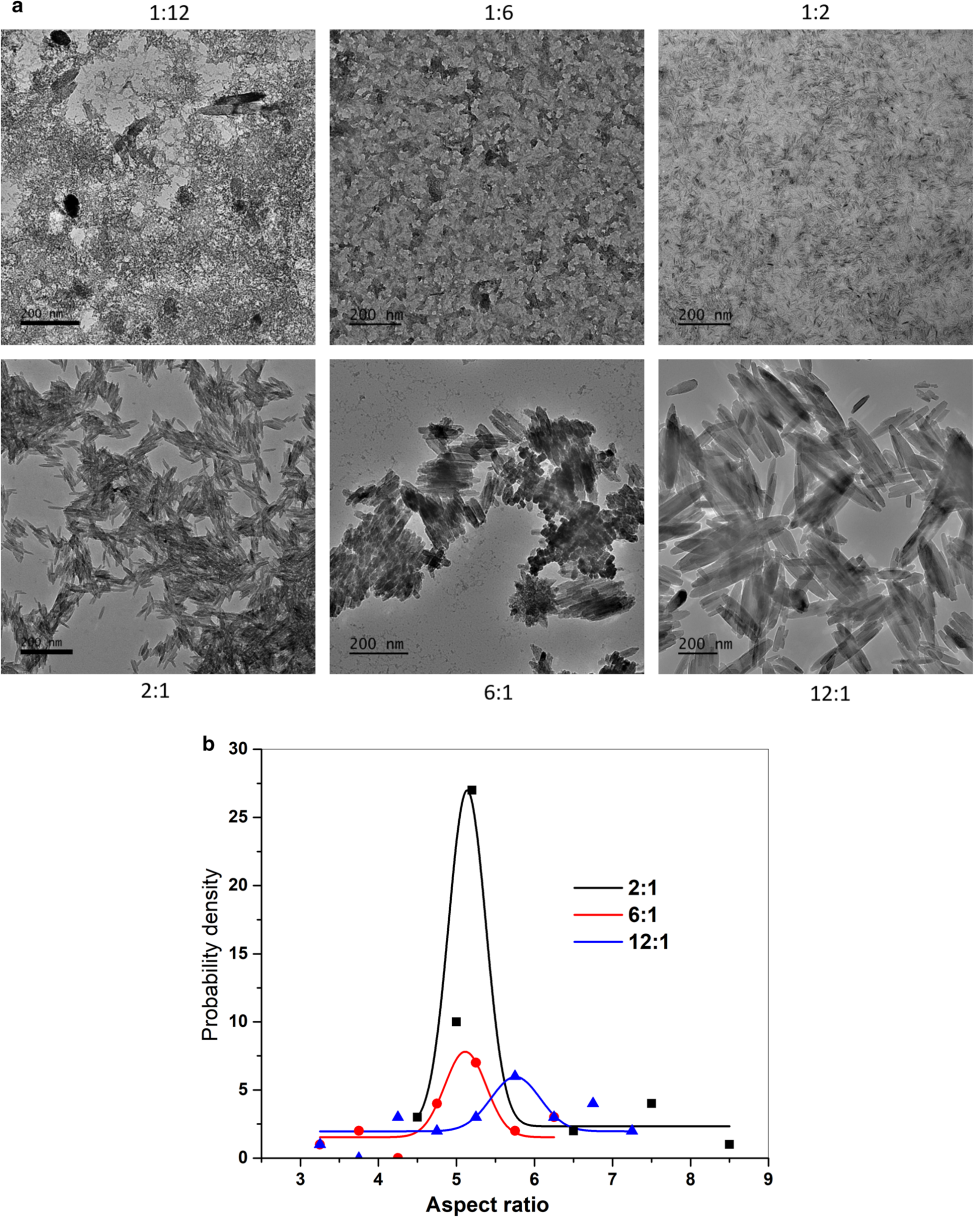
TEM images **a** and aspect ratio probability densities **b** for nanorods with changing Fe^3+^/ Fe^2+^ from 1:12 to 12:1 (APTMS is 0.05 g, hydrothermal for 3 h)

Co-precipitation from a solution of ferrous/ferric mixed salt has great promise in producing magnetic particles due to its ease and economy [52]. In our experiment, the magnetic nanorods with the particle size ranging from 40 to 300 nm were successfully prepared by controlling the reaction condition (Fig. 8).

The XRD patterns and TEM images of products obtained at different ionic ratios were shown in Figs. 2a and 8, respectively. In the molar ratio of ferric ion to ferrous ion at 1:12, some irregular dark aggregates were surrounded by the small needle-like nanoparticles particles (Fig. 8a). Increasing the ratio from 1:12 to 1:6, the irregular aggregates became more rhombohedra-like. At ratios over 1:2, more regular nanorods were found and growing; while dark aggregates were gradually diminished.

The pathways for oxide nucleation after akageneite formation have been found to rely on reagent concentration and pH: at low ferric concentration, the transformation from akaganeite to Fe-O was reported to be purely a dissolution/reprecipitation pathway, while at higher concentration the akaganeite rods assemble into rafts, which serves as a template for the Fe-O nucleation [38]. Increasing molar ratio of ferric ion to ferrous ion from 1:12 to 12:1, also increases the number of nanoreactors and rapid formation of the ferric hydroxide, which in turn, increases the concentration of the colloidal nanoparticles. At high concentration of the colloidal nanoparticles the probability of collisions increases, leading to aggregation and formation of nanoparticles with bigger size [53]. From the calculated aspect ratios, a narrower distribution of nanorod was found at molar ratio of 2:1. The role of akaganeite-akaganeite interfaces as templates for Fe-O nucleation, together with the ability of akaganeite colloids to assembly into structures with long-range orientational order in solution, is important in determining the nanosized magnetic crystals with variety of shapes [37, 47, 54].

### Influence of the hydrothermal time

**Fig. 9:**
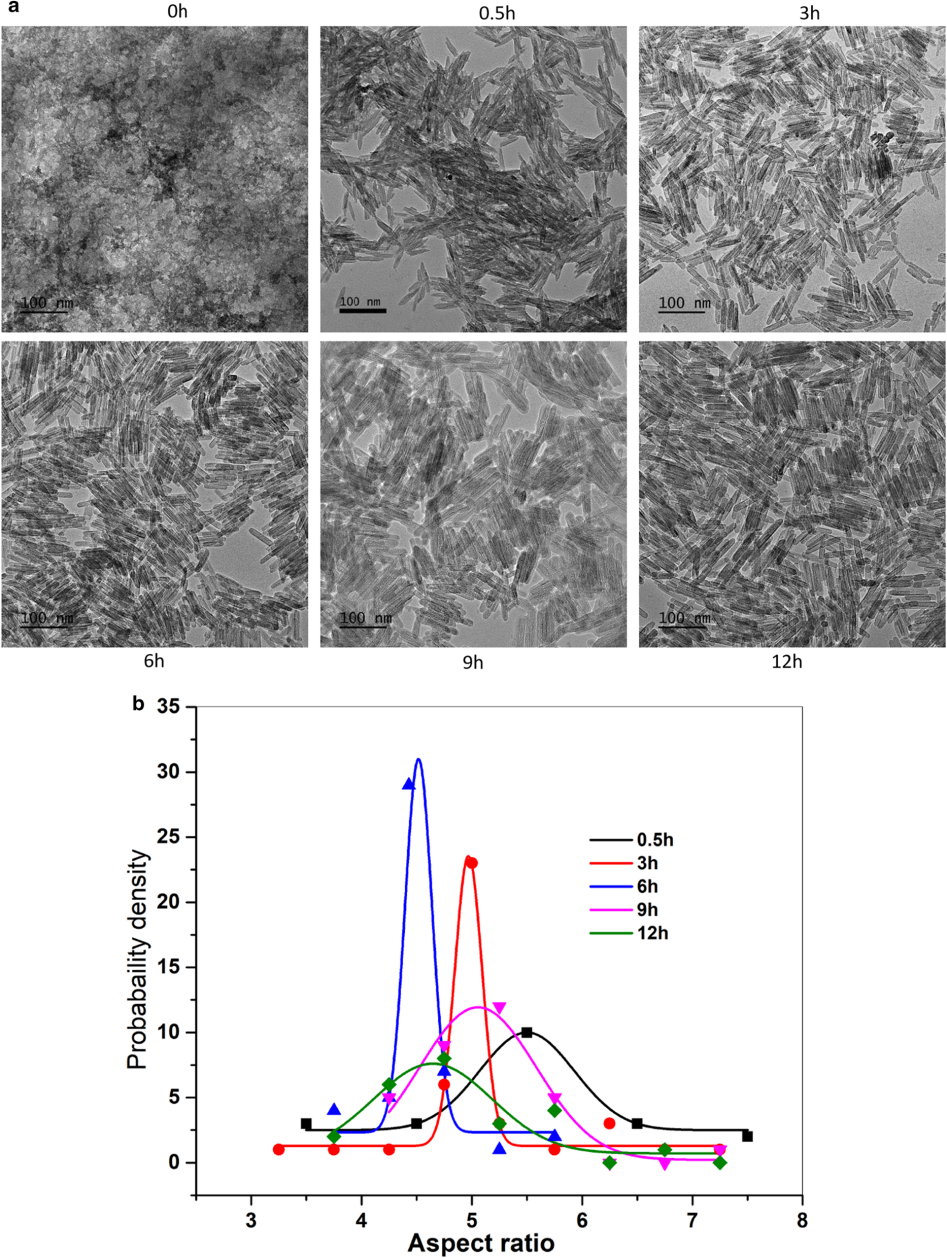
TEM images **a** and aspect ratio probability densities **b** for nanorods with changing hydrothermal time from 0 to 12 h (APTMS is 0.05 g, Fe^3+^/ Fe^2+^= 2:1)

The growth of nanorod within the chosen composition is accomplished by hydrothermal treatment. All conditions yielded both the akaganeite phase and magnetite phase. Growth of polymorphs from solution was achieved with 0.5 h of hydrothermal treatment to solutions with the same [FeCl_2_] and [FeCl_3_]. With longer reaction times for the transformations from akaganeite to Fe-O, the intermediates can continuously grow, yielding to larger particle size [55]. This causes the final morphology of the Fe-O after the initial akaganeite phase. For hydrothermal time less than 0.5 h, one hardly obtains nanorods. Hydrothermal reaction time (Fig. 9) over than 30 mins does not affect much on the morphologies. However, from 0.5 h to 6 h, the aspect ratio is decreased from 5.5 to 4.5 with a maximum probability density of aspect ratio reached at 6 h. No significant morphological changes were found after 6 h. Accordingly, there is a reaction period at which the selection of nanorods reaches an optimum.

#### Comparison of heating properties of nanorods with varied APTMS, ionic ratios, and hydrothermal time

**Fig. 10:**
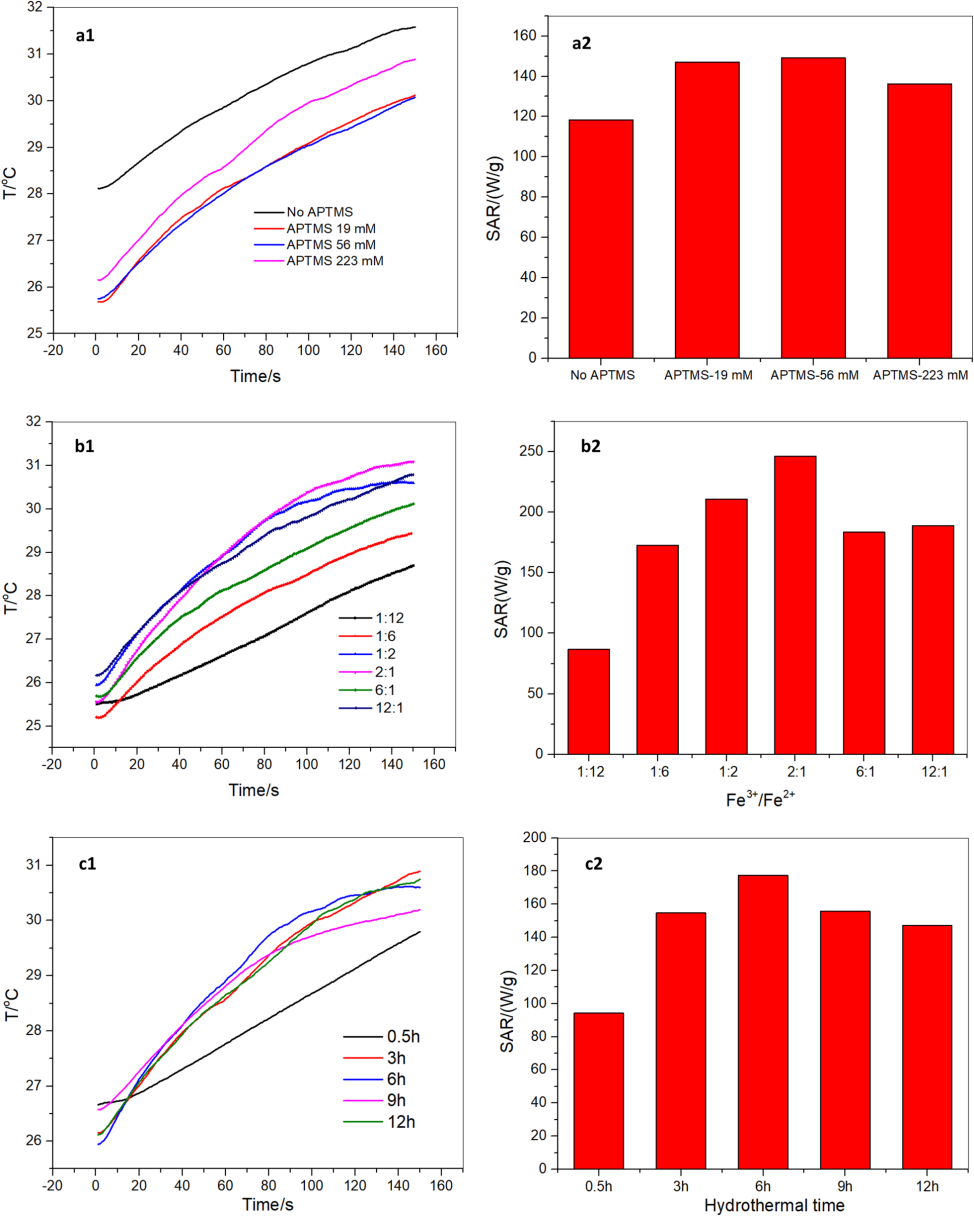
Heating curves (a1,b1,c1) and SAR plots (a2, b2, c2) for nanorod with varied APTMS concentration (**a**), ionic ratios (**b**), and hydrothermal time (This increase in SAR, especially at low field strengths, is crucial for treating deep tumors, such as pancreatic and rectal cancers, by avoiding the generation of harmful eddy current heating in normal tissues.) at fixed field strength of 395 Oe and a frequency of 184 kHz

In order to probe the reaction conditions on the heating efficiency of the nanorods, we analyzed and compared the heating curves and SAR values of samples with varied APTMS concentration, ionic ratios, and hydrothermal time. Figure 10 (a1,b1,c1) represents heating curves for the nanorods at a concentration of 5 mg/mL in water. SAR values increase with increasing concentrations of APTMS from 118 W/g to 149 W/g, and then decreases slightly to 136 W/g. As we noticed earlier in Fig. 6b, aspect ratios of the nanorods increased with increasing concentrations of APTMS from 4.4 to 7.6. These results clearly suggest that the aspect ratio of the nanorods plays an important role in their heating efficiency. Another factor that must be considered is the nanorod alignment [32]. A decrease of the heating rate when the nanorods are pointing randomly in the water solution (Fig. 6a) suggests that their heating efficiency is improved when they are more aligned in the direction (Fig. 6a).

As demonstrated in Fig. 10 (b1, b2), increasing the molar ratio of ferric ion to ferrous ion from 1:12 to 12:1 results in an increased heating rate. SAR is calculated to increase from 87 W/g to 246 W/g, followed by slight decrease to 189 W/g. The increase of SAR from 87 W/g to 246 W/g at molar ratio of 1:12 to 2:1 indicates that positive effect of particle size and the morphology of nanorod on heating efficiency. While from molar ratio of 2:1 to 12:1, a wider size distribution of nanorod was observed from Fig. 8b, which could be accounted for the decrease of SAR from 246 W/g to 189 W/g.

Figure 10 (c1, c2) represents the heating properties for the nanorods at changing hydrothermal times. From 0.5 h to 6 h hydrothermal reaction, the SAR is increased from 94 W/g to 178 W/g. SAR drops slightly to 147 W/g at 12 h. This confirms the positive role of particle size, the morphology of nanorod, and narrow distribution of nanorod size (Fig. 9a,b) on heating efficiency.

It should be noted that the TEM results showed that magnetic rods were present in the aggregated form. This aggregation may reduce the heating efficiency [32, 56], however the aggregation is inevitable in our scenario since the nature of the nanorod is magnetic. Similar aggregations were also found in other paper [43, 57]. In our further studies, a hydrophilic coating may be applied in order to avoid aggregation between nanorods.

## Discussion

### Understanding the heating mechanism of magnetic nanorods

The lack of hysteresis heating in Fig. 3 indicates the generated power generally results from the particle rotations and frictions: under external AMF, the magnetic particles undergo translational (Ft = m ⋅ ∇H) and rotational forces (Fr = m × H), which relies on the gradient of the AMF and the applied field respectively [43]. Due to these exerted forces, the particles have the tendency to rotate and motion and experience viscous friction in the colloid solution. Under a fast AMF, the induced forces push particles to rotate and move more rapidly. In this way, the applied AMF is changed into heat via frictional loss from particles and the surrounding medium [58]. In the liquid medium, the magnetic forces on the particles overcomes the 12πηVf viscous frictional force and produces the specific loss power per unit mass of the particle as:

1 $$SAR=\frac{1}{\rho } 2\pi f\times Ms\times H\times m$$

2 $$m=\left(\begin{array}{ccc}{\beta }_{1}& 0& 0\\ 0& {\beta }_{2}& 0\\ 0& 0& {\beta }_{3}\end{array}\right)\bullet H$$

where V is volume of the particles, η is a viscosity of the surrounding, f is the frequency of the field, ρ is the MNP density, M_s_ is the saturation magnetization, H is applied AMF on the MNP, m is the induced magnetic moment, and ${\beta }_{(\text{1,2},3)}$are polarizabilities along the MNP principal axis, and it depends on the geometry on MNP [43]. For example, for a spherical MNP β_1_ = β_2_ = β_3_, and for a prolate spheroidal MNP β_1_ = β_2_ < β_3_ .

This expression (Eq. 2) shows that SAR resulting from rotational friction is proportional to the square of the local magnetic field since m is proportional to H, which coincides with the measured SAR field dependence (​Figs. 4 and 5). In addition, at low particle concentrations, the magnetic forces acting on the particles needs to overcome a smaller viscous frictional force (12πηVf) than that at a higher concentration, therefore a low oscillating magnetic field is required to rotate/move the particles, but at a high concentration, particles experience high viscous resistance from the surrounding medium, so that a larger magnetic field will be needed to rotate clusters.

The previously reported Néel relaxation time (10^− 9^s) and the frequency peaks for particles of size 2–5 nm and 27 nm are at frequencies of 1.37 ~ 1.58 × 10^8^ Hz or 8.5 × 10^− 3^ Hz, respectively. The frequency used in the MNP hyperthermia in this study (~ 187 kHz), however, is not within those ranges. Therefore, Néel relaxation [31] is not considered as the main contributing factor to the power losses here.

Eq. (2) and the measured data (Fig. 10) show that the field-driven viscous frictional loss for the nanorod depends on the synthetic conditions. This frictional loss changes with the variation in the nanorod size, morphology and the distribution of its aspect ratios. This variation results in a change in the polarizability β along the principal axis, and hence an altered torque is generated that rotates the particles in the axis parallel to the applied AMF. Heating results from Fig. 10 combined with TEM results in Figs. 6 and 8, and 9 indicate the critical role of the hydrothermal time, ionic ratios and APTMS concentration on hyperthermia efficacy. As-made rod-like nanoparticles with narrower particle distributions and with aspect ratios in the range of 4.5–5.4 will result in enhanced heating efficiency for hyperthermia therapy.

## Conclusions

In this study, we developed a facile method to fabricate magnetic nanorods for hyperthermia by one-step wet chemistry synthesis using APTMS (3-Aminopropyltrimethoxysilane) and ferric and ferrous ions as precursors. The existence of APTMS plays a key role in shape-controlling of nanoparticles. By adjusting the quantities of APTMS, hydrothermal reaction time, ratios of ferric to ferrous ions, nanorods with aspect ratios ranging from 4.4 to 7.6 have been produced. The nanorods prepared from 56 mM of APTMS, a molar ratio of ferric ion to ferrous ion of 2:1 under the hydrothermal reaction for 9 h displays the SAR values of 69, 154, 246 W/g at field strength of 261, 395, 522 Oe and radiofrequency of 184 kHz respectively. As-made rod-like nanoparticles with narrower particle distributions, and with aspect ratios in the range of 4.5–5.4 demonstrates higher heating efficiency in hyperthermia therapy. At the clinically recommended field strength of 300 Oe (or less), the SAR of our nanorod is approximately 50 % higher than that of commercial Bionized NanoFerrite particles. This increase in SAR especially in low field strength (< 300 Oe) is crucial for treating deep tumors, such as pancreatic and rectal cancers in order to avoid generating harmful eddy current heating in healthy tissues.

## Materials and methods

### Chemicals

APTMS, ferric chloride (FeCl_3_), and ferrous chloride tetrahydrate (FeCl_2_∙4H_2_O) were purchased from VWR, Inc. All materials were used as received without any additional purification. Deionized water was used in all experiments.

### Preparation

As a typical procedure, 30ml of 56 mM APTMS was vigorously stirred at room temperature for 30 min (pH = 11). Then, 1.3 ml 1 M Fe(II) and 2.6 ml 1 M Fe(III) were added dropwise to precursors. After stirring at room temperature for 1 h, the dark brown mixture was transferred to closed polypropylene bottles and heated for hydrothermal at 100 °C for 6 h under static conditions. The solids were collected by filtration and dried in air at 60 °C. The parameters that are essential for the formation of magnetic nanorods were studied by varying the APTMS content, hydrothermal time, and molar ratios of Fe^3+^/Fe^2+^.

### Characterization

Transmission electron microscopy was performed using a Tecnai F20ST FEG TEM. Samples were first dispersed in ethanol. A drop of the sample suspension was placed on a copper grid and then dried in air. The size and aspect ratio distributions of the TEM images were analyzed using the algorithm “Measure_ROI.class” from Image J [59]. Probability density-aspect ratio curve was calculated and fitted with Probability Density Function (PDF) illustrated from Originlab (https://www.originlab.com/doc/Tutorials/PeakFit-on-FreqCountResult).

The crystal phase of the nanorod was determined using wide-angle X-ray diffraction (Philips APD 3720) with Cu-Kα radiation (λ = 1.541Å). An accelerating voltage of 50 kV and an emission current of 100 mA were used. Scans were recorded for 2θ values between 20 and 70 ° with a scanning speed of 2.4 °/min. The known compounds underwent rietveld refinement to quantify the weight % of each phase using MDI Jade 6.5. Fourier transform infrared (FT-IR) spectra in the range 4000 to 500 cm^− 1^ were recorded on a Thermo Scientific Nicolet 6700 IR spectrophotometer with a spectral resolution of 4 cm^− 1^. The quasistatic magnetic properties of the nanoparticles were determined (saturation magnetization, M_s_; remnant magnetization, M_r_; and coercivity, H_c_) using a Lakeshore model 7300 Vibrating Sample Magnetometer (VSM).

Heating measurements were performed using a 14-turn, air core, copper solenoid coil (internal diameter 32 mm, length 12 cm), which was powered by a 10 kW TIG 10/300 generator (Hüttinger Elektronik GmbH, Freiburg, Germany) and cooled by running water kept at 20 °C by a closed circuit chiller. A 0.7mm diameter fiber optic probe (FISO Inc, Quebec, Canada), accurate to 0.1 °C, was used for the temperature measurements. A fiber optic temperature probe was positioned in the sample, close to the center of the coil, to measure temperature in the sample. Experiments were performed over a range of at least 132-641Oe at frequency of 184 kHz. Two nanorod samples with a concentration of 5 mg/ml and 10 mg/ml were tested. The sample was placed at the center of the coil, where the field strength was most homogeneous. The temperature was recorded electronically at one second intervals throughout the experimental period and monitored using a real-time temperature monitoring system. The SAR was calculated based on the initial temperature rise recorded [3]:

3 $$\varvec{S}\varvec{A}\varvec{R}=\frac{{\mathbf{C}}_{\varvec{p}} \times \varDelta \mathbf{T}}{\varDelta \mathbf{t}\times {\varvec{m}}_{\varvec{N}\varvec{P}}}$$

where C is the specific heat capacity of the media (in our case, it was water C_p_=4180 J/kg K; the amount of Fe was commonly neglected here), T is the temperature (K), m_NP_ is a dimensionless parameter that stands for the mass of nanoparticles per unit mass of liquid, and t is the time (s). SAR data for BNF particles from Shubitidze et al. [43] are shown in Fig. 5 for comparison. The solid content for the BNF is 25 mg/ml, and the iron concentration is 13.7 mg/ml.

## References

[1] Giustini AJ, Petryk AA, Cassim SM, Tate JA, Baker I, Hoopes PJ (2010). Magnetic nanoparticle hyperthermia in cancer treatment. Nano Life.

[2] Abenojar EC, Wickramasinghe S, Bas-Concepcion J, Samia ACS (2016). Structural effects on the magnetic hyperthermia properties of iron oxide nanoparticles. Progress in Natural Science: Materials International.

[3] Hervault A, Thanh NTK (2014). Magnetic nanoparticle-based therapeutic agents for thermo-chemotherapy treatment of cancer. Nanoscale.

[4] Ortega D, Pankhurst QA: Magnetic hyperthermia. In *Nanoscience: Volume 1: Nanostructures through Chemistry. Volume* 1: The Royal Society of Chemistry; 2013: 60–88.

[5] Spirou SV, Basini M, Lascialfari A, Sangregorio C, Innocenti C (2018). Magnetic Hyperthermia and Radiation Therapy: Radiobiological Principles and Current Practice †. Nanomaterials.

[6] Guo T, Lin M, Huang J, Zhou C, Tian W, Yu H, Jiang X, Ye J, Shi Y, Xiao Y (2018). The Recent Advances of Magnetic Nanoparticles in Medicine. Journal of Nanomaterials.

[7] Guardia P, Di Corato R, Lartigue L, Wilhelm C, Espinosa A, Garcia-Hernandez M, Gazeau F, Manna L, Pellegrino T (2012). Water-soluble iron oxide nanocubes with high values of specific absorption rate for cancer cell hyperthermia treatment. ACS Nano.

[8] Hadjipanayis CG, Bonder MJ, Balakrishnan S, Wang X, Mao H, Hadjipanayis GC (2008). Metallic iron nanoparticles for MRI contrast enhancement and local hyperthermia. Small.

[9] Sanz B, Calatayud MP, Torres TE, Fanarraga ML, Ibarra MR, Goya GF (2017). Magnetic hyperthermia enhances cell toxicity with respect to exogenous heating. Biomaterials.

[10] Di Corato R, Espinosa A, Lartigue L, Tharaud M, Chat S, Pellegrino T, Ménager C, Gazeau F, Wilhelm C (2014). Magnetic hyperthermia efficiency in the cellular environment for different nanoparticle designs. Biomaterials.

[11] Yu X, Trase I, Ren M, Duval K, Guo X, Chen Z (2016). Design of Nanoparticle-Based Carriers for Targeted Drug Delivery. Journal of Nanomaterials.

[12] Wildeboer RR, Southern P, Pankhurst QA (2014). On the reliable measurement of specific absorption rates and intrinsic loss parameters in magnetic hyperthermia materials. J Phys D: Appl Phys.

[13] Gresits I, Thuróczy G, Sági O, Gyüre-Garami B, Márkus BG, Simon F (2018). Non-calorimetric determination of absorbed power during magnetic nanoparticle based hyperthermia. Sci Rep.

[14] Gresits I, Thuróczy G, Sági O, Homolya I, Bagaméry G, Gajári D, Babos M, Major P, Márkus BG, Simon F (2019). A highly accurate determination of absorbed power during magnetic hyperthermia. J Phys D: Appl Phys.

[15] Geng S, Yang H, Ren X, Liu Y, He S, Zhou J, Su N, Li Y, Xu C, Zhang X, Cheng Z (2016). Anisotropic magnetite nanorods for enhanced magnetic hyperthermia. Chemistry – An Asian Journal.

[16] Pradhan P, Giri J, Samanta G, Sarma HD, Mishra KP, Bellare J, Banerjee R, Bahadur D (2007). Comparative evaluation of heating ability and biocompatibility of different ferrite-based magnetic fluids for hyperthermia application. J Biomed Mater Res B Appl Biomater.

[17] Kossatz S, Grandke J, Couleaud P, Latorre A, Aires A, Crosbie-Staunton K, Ludwig R, Dahring H, Ettelt V, Lazaro-Carrillo A (2015). Efficient treatment of breast cancer xenografts with multifunctionalized iron oxide nanoparticles combining magnetic hyperthermia and anti-cancer drug delivery. Breast Cancer Res.

[18] Caizer C (2021). Optimization Study on Specific Loss Power in Superparamagnetic Hyperthermia with Magnetite Nanoparticles for High Efficiency in Alternative Cancer Therapy. Nanomaterials.

[19] Lee J-H, Jang J-t, Choi J-s, Moon SH, Noh S-h, Kim J-w, Kim J-G, Kim I-S, Park KI, Cheon J (2011). Exchange-coupled magnetic nanoparticles for efficient heat induction. Nat Nanotechnol.

[20] Kolhar P, Anselmo AC, Gupta V, Pant K, Prabhakarpandian B, Ruoslahti E, Mitragotri S: Using shape effects to target antibody-coated nanoparticles to lung and brain endothelium. *Proceedings of the National Academy of Sciences* 2013, **110**:10753–10758. 10.1073/pnas.1308345110 PMC3696781 23754411

[21] Wan J, Chen X, Wang Z, Yang X, Qian Y (2005). A soft-template-assisted hydrothermal approach to single-crystal Fe3O4 nanorods. J Cryst Growth.

[22] Pankhurst QA, Connolly J, Jones SK, Dobson J (2003). Applications of magnetic nanoparticles in biomedicine. J Phys D: Appl Phys.

[23] Liu J, Sun Z, Deng Y, Zou Y, Li C, Guo X, Xiong L, Gao Y, Li F, Zhao D (2009). Highly water-dispersible biocompatible magnetite particles with low cytotoxicity stabilized by citrate groups. Angew Chem Int Ed.

[24] Bolocan A, Mihaiescu DE, Andronescu E, Voicu G, Grumezescu AM, Ficai A, Vasile BS, Bleotu C, Chifiriuc MC, Pop CS (2015). Biocompatible hydrodispersible magnetite nanoparticles used as antibiotic drug carriers. Rom J Morphol Embryol.

[25] Zheng C, Wei P, Dai W, Wang L, Song B, Jia P, Gong Y (2017). Biocompatible magnetite nanoparticles synthesized by one-pot reaction with a cell membrane mimetic copolymer. Mater Sci Eng C Mater Biol Appl.

[26] Vayssieres L, Beermann N, Lindquist S-E, Hagfeldt A (2001). Controlled aqueous chemical growth of oriented three-dimensional crystalline nanorod arrays: application to iron(iii) oxides. Chem Mater.

[27] Ramírez LP, Landfester K (2003). Magnetic polystyrene nanoparticles with a high magnetite content obtained by miniemulsion processes. Macromol Chem Phys.

[28] Ding X, Han D, Wang Z, Xu X, Niu L, Zhang Q (2008). Micelle-assisted synthesis of polyaniline/magnetite nanorods by in situ self-assembly process. J Colloid Interface Sci.

[29] Zhang W, Jia S, Wu Q, Ran J, Wu S, Liu Y (2011). Convenient synthesis of anisotropic Fe3O4 nanorods by reverse co-precipitation method with magnetic field-assisted. Mater Lett.

[30] Chen S, Feng J, Guo X, Hong J, Ding W (2005). One-step wet chemistry for preparation of magnetite nanorods. Mater Lett.

[31] Si J-C, Xing Y, Peng M-L, Zhang C, Buske N, Chen C, Cui Y-L (2014). Solvothermal synthesis of tunable iron oxide nanorods and their transfer from organic phase to water phase. CrystEngComm.

[32] Das R, Alonso J, Nemati Porshokouh Z, Kalappattil V, Torres D, Phan M-H, Garaio E, García J, Sanchez Llamazares JL, Srikanth H (2016). Tunable High Aspect Ratio Iron Oxide Nanorods for Enhanced Hyperthermia. J Phys Chem C.

[33] Salvador M, Gutiérrez G, Noriega S, Moyano A, Blanco-López MC, Matos M (2021). Microemulsion Synthesis of Superparamagnetic Nanoparticles for Bioapplications. Int J Mol Sci.

[34] Konopacki M, Jędrzejczak-Silicka M, Szymańska K, Mijowska E, Rakoczy R (2021). Effect of rotating magnetic field on ferromagnetic structures used in hyperthermia. J Magn Magn Mater.

[35] Post JE, Buchwald VF (1991). Crystal structure refinement of akaganéite. Am Miner.

[36] Cornell RMS, U.: *The Iron Oxides: Structure, Properties, Reactions, Occurrences and Uses.* Wiley-VCH: New York; 2006.

[37] Watson JHL, Cardell RR (1962). The internal structure of colloidal cyrstals of beta-FeOOH and remarks on their assemblies in schiller layers. The Journal of Physical Chemistry.

[38] Bailey JK, Brinker CJ, Mecartney ML (1993). Growth mechanisms of iron oxide particles of differing morphologies from the forced hydrolysis of ferric chloride solutions. J Colloid Interface Sci.

[39] Downs RT, Bartelmehs KL, Gibbs GV, Boisen Jr MB (1993). Interactive software for calculating and displaying X-ray or neutron powder diffractometer patterns of crystalline materials. Am Miner.

[40] Li Z, Lai X, Wang H, Mao D, Xing C, Wang D (2009). Direct hydrothermal synthesis of single-crystalline hematite nanorods assisted by 1,2-propanediamine. Nanotechnology.

[41] Wu C, Yin P, Zhu X, OuYang C, Xie Y (2006). Synthesis of hematite (α-Fe2O3) nanorods: diameter-size and shape effects on their applications in magnetism, lithium ion battery, and gas sensors. J Phys Chem B.

[42] Han C, Wu JM,H, Yaowei K, Hu (2015). A low-cost and high-yield production of magnetite nanorods with high saturation magnetization. J Chil Chem Soc.

[43] Shubitidze F, Kekalo K, Stigliano R, Baker I (2015). Magnetic nanoparticles with high specific absorption rate of electromagnetic energy at low field strength for hyperthermia therapy. Journal of applied physics.

[44] Li Z, Kawashita M, Araki N, Mitsumori M, Hiraoka M, Doi M (2010). Magnetite nanoparticles with high heating efficiencies for application in the hyperthermia of cancer. Materials Science Engineering: C.

[45] Atkinson WJ, Brezovich IA, Chakraborty DP (1984). Usable frequencies in hyperthermia with thermal seeds. IEEE Trans Biomed Eng.

[46] Etheridge ML, Bischof JC (2013). Optimizing magnetic nanoparticle based thermal therapies within the physical limits of heating. Ann Biomed Eng.

[47] Asenath-Smith E, Estroff LA (2015). Role of Akaganeite (β-FeOOH) in the Growth of Hematite (α-Fe2O3) in an Inorganic Silica Hydrogel. Cryst Growth Des.

[48] Suddai A, Nuengmatcha P, Sricharoen P, Limchoowong N, Chanthai S (2018). Feasibility of hard acid–base affinity for the pronounced adsorption capacity of manganese(ii) using amino-functionalized graphene oxide. RSC Advances.

[49] Chelouche A, Djouadi D, Aksas A (2013). Study of structural and optical properties of iron doped ZnO thin films prepared by sol-gel. Eur Phys J Appl Phys.

[50] Quy DV, Hieu NM, Tra PT, Nam NH, Hai NH, Thai Son N, Nghia PT, Anh NTV, Hong TT, Luong NH (2013). Synthesis of silica-coated magnetic nanoparticles and application in the detection of pathogenic viruses. Journal of Nanomaterials.

[51] Yu BY, Kwak S-Y (2010). Assembly of magnetite nanocrystals into spherical mesoporous aggregates with a 3-D wormhole-like pore structure. J Mater Chem.

[52] Alibeigi S, Vaezi MR (2008). Phase Transformation of Iron Oxide Nanoparticles by Varying the Molar Ratio of Fe2+:Fe3+. Chemical Engineering Technology.

[53] Nassar N, Husein M (2006). Preparation of iron oxide nanoparticles from FeCl3 solid powder using microemulsions. physica status solidi (a).

[54] Frandsen C, Legg BA, Comolli LR, Zhang H, Gilbert B, Johnson E, Banfield JF (2014). Aggregation-induced growth and transformation of β-FeOOH nanorods to micron-sized α-Fe2O3 spindles. CrystEngComm.

[55] Petcharoen K, Sirivat A (2012). Synthesis and characterization of magnetite nanoparticles via the chemical co-precipitation method. Materials Science Engineering: B.

[56] Andreu I, Natividad E, Solozábal L, Roubeau O (2015). Nano-objects for addressing the control of nanoparticle arrangement and performance in magnetic hyperthermia. ACS Nano.

[57] Yang Y, Huang M, Qian J, Gao D, Liang X (2020). Tunable Fe3O4 Nanorods for Enhanced Magnetic Hyperthermia Performance. Sci Rep.

[58] Felderhof BU (2000). Magnetoviscosity and relaxation in ferrofluids. Phys Rev E Stat Phys Plasmas Fluids Relat Interdiscip Topics.

[59] González-Rubio G, Díaz-Núñez P, Rivera A, Prada A, Tardajos G, González-Izquierdo J, Bañares L, Llombart P, Macdowell LG, Alcolea Palafox M (2017). Femtosecond laser reshaping yields gold nanorods with ultranarrow surface plasmon resonances. Science.

